# Treatment trajectories among patients with musculoskeletal disorders in Norway – a register-based cohort study over 2 years

**DOI:** 10.1080/02813432.2026.2633751

**Published:** 2026-02-25

**Authors:** Mari Kristine Tyrdal, Flavie Perrier, Cecilie Røe, Bård Natvig, Astrid Klopstad Wahl, Marit Bragelien Veierød, Hilde Stendal Robinson

**Affiliations:** a Department of Public Health Science and Interdisciplinary Health Sciences, University of Oslo, Oslo, Norway; b Oslo Centre for Biostatistics and Epidemiology, Department of Biostatistics, Institute of Basic Medical Sciences, University of Oslo, Oslo, Norway; c Department of Physical Medicine and Rehabilitation, Oslo University Hospital, Oslo, Norway; d Department of General Practice, Institute of Health and Society, Institute of Clinical Medicine, University of Oslo, Oslo, Norway

**Keywords:** Register data, general practitioner, physiotherapy, primary healthcare, specialist healthcare

## Abstract

**Objective:**

Explore treatment trajectories over a two-year follow-up among patients with musculoskeletal disorders (MSDs) in Norway and investigate the associations with education and country of origin.

**Design:**

Register-based cohort study.

**Settings:**

Data were obtained from three national registers, the Norwegian Control and Payment of Health Reimbursements Database (KUHR), the Norwegian Patient Registry (NPR) and Statistics Norway (SSB).

**Subjects:**

Patients diagnosed with MSDs in 2015 in primary healthcare according to the International Classification of Primary Care, second edition (ICPC-2).

**Main outcome measures:**

Treatment trajectory classes of healthcare use over a 2-years follow-up were generated using group-based multi-trajectory modelling, for all patients registered with MSDs and three common diagnoses: spine pain, osteoarthritis and fibromyalgia.

**Results:**

From the analysis, four treatment trajectory classes of healthcare use were identified for MSD patients overall; low (75%), stable (10%), descending (9%) and high use (5%). The pattern was almost similar for the three diagnostic groups. High use was primarily caused by consistent high use of physiotherapy. For MSD patients overall, low education increased the probability of being in the low and stable use classes, while high education increased the probability of being in the descending use class. Patients with Norway as country of origin, were less likely to be in the class with low use and more likely to be in the class with descending use.

**Conclusion:**

Using nationwide register data and group-based multi-trajectory modelling show that about 75% of all patients with MSD, consulting primary healthcare in Norway, were classified as low users of healthcare services and 5% were classified as high users. Overall, both education and country of origin were associated with the treatment trajectory classes. However, high use was not associated with neither education nor country of origin.

## Introduction

Musculoskeletal disorders (MSDs) pose a global health challenge, with heavy burdens on the healthcare systems [1,2]. However, a small proportion of patients with MSDs accounts for the majority of the healthcare resources [3–6]. MSDs are treated in both primary and specialist healthcare, yet there is uncertainty regarding the most effective treatment pathways for several conditions. This may contribute to developing persistent pain and long-lasting need for care [7]. With an ageing population, reduced resources and stricter prioritization in the years to come, it is important to improve treatment strategies. To achieve this, a better understanding of the existing treatment trajectories for various MSDs could be essential.

MSDs include diagnostic groups with diverse characteristics and pathogenesis [8]. Spine pain, fibromyalgia (FM) and osteoarthritis (OA) are three common MSDs [9] with different symptoms, expected course and healthcare use. About 20% of the healthcare contacts in primary healthcare (PHC) are because of spine pain [9] and this is also the leading cause of years lost to disability worldwide [10]. Fibromyalgia, characterized by persistent and widespread pain, is more frequent among women than men, and 6% prevalence was reported among women in Norway in 1991–2007 [11,12]. OA implies degeneration of articular cartilage and is increasing in the ageing population. Based on healthcare register data from 2008 to 2017, the period prevalence of OA in the Nordic countries was nearly 14% [13]. One common characteristic of the three diagnoses is the tendency to become long-lasting. However, we have limited knowledge about healthcare use over time and potential differences within and between these diagnostic groups.

The general practitioners (GPs) are the main health provider for MSD patients and gatekeepers for referrals to other healthcare services in Norway [14]. Moreover, physiotherapists (PTs) in PHC and physicians in specialist healthcare (SHC) are commonly consulted. The latter is more often due to long-lasting MSDs and the need for multidisciplinary follow-up and specialist declarations [7,15]. About 50% of patients with long-lasting MSDs in Norway are referred to PT and/or SHC within two years [16]. Previous studies have identified subgroups with diverse use of healthcare services [4,5,17–19]. However, these studies differed in terms of the disorders investigated, sample sizes, statistical methods and duration of follow-up, resulting in the identification of three to seven distinct treatment trajectories classes. We found no studies exploring treatment trajectories in PHC and SHC among patients with spine pain and FM, nor using nationwide registers encompassing the entire population. Follow-up time in the literature ranged from 1 to 10 years, with most patients consulting their preferred healthcare providers within the first few years. In the light of these findings and to address existing knowledge gaps, we aimed to explore treatment trajectories over a two-year period.

About 90% of all Norwegian healthcare services are publicly funded, and every citizen has the same right to healthcare services [14]. Despite the intention of equality, studies have revealed differences in healthcare use due to age, sex, socioeconomic status, education and ethnicity [20,21]. Individuals with lower socioeconomic status utilized healthcare services more frequently, while consulting a specialist was associated with higher education [6,20]. Moreover, studies have found that immigrants consulted the GP more seldom compared with natives and were more prone to visit the emergency room instead of the GP [22,23]. To provide and ensure equitable healthcare services, tailored to patients’ individual needs for all citizens can be challenging. Cultural and language barriers, health literacy and knowledge of rights can affect how people seek healthcare [24–26]. Education and country of origin are therefore important factors to consider when examining treatment trajectories and healthcare use.

Using nationwide, population-based registries in Norway, we explore healthcare use among patients with MSDs over a two-year period. Our aim was to identify treatment trajectories in general and in three common diagnostic groups (spine pain, FM and OA), and to investigate associations with education and country of origin.

## Materials and methods

We used data from three national health registers: the Norwegian Control and Payment of Health Reimbursement Database (KUHR), the Norwegian Patient Registry (NPR) and Statistics Norway (SSB) [27]. The unique personal identification number of all citizens enables register linkage. The study period was January 2015 through December 2017.

The Regional Committee for Medical and Health Research Ethics in Norway approved this project (2018-1280-1).

### Data from primary healthcare

KUHR is the national database containing reimbursement claims from publicly funded healthcare services, including GPs and PTs, in PHC in Norway, handled by the Norwegian Directorate of Health. The information in KUHR is obtained directly from electronic administrative patient registration systems in the clinics, giving practically 100% data completeness [28]. Each patient-related contact is registered with demographics, healthcare professional information, reimbursement rates, and diagnostic codes. KUHR uses the International Classification of Primary Care, second edition (ICPC-2) as a diagnostic tool where L-diagnoses refer to MSD [29].

### Data from specialist healthcare

NPR includes information about patient activity in SHC and is an administrative database of records reported by the publicly funded hospitals and publicly funded outpatient specialist clinics in Norway. Demographic characteristics, diagnosis and type of contact (outpatient, inpatient or day patient) are registered for each contact. Diagnoses are coded according to the International Classification of Diseases, 10^th^ revision (ICD-10), where M-diagnoses refer to MSD [29].

### Linkage of register data

All patients with L-diagnoses and registered with GP and/or PT consultations in KUHR in 2015, were identified and linked with data from the NPR registered with M-diagnoses for information about healthcare services in SHC, and with SSB for level of education and country of origin. Date of the first consultation in 2015 was set as baseline. Patients with a non-physical consultation, identified by the reimbursement rate, were not included. Such consultations were less common during the follow-up period but have increased during and after the COVID-19 pandemic. We excluded patients registered with MSD diagnoses in KUHR and/or NPR during the 12 months prior to the first contact in 2015 and patients <18 years. Patients were classified with spine pain, FM and OA if these diagnoses were recorded both at the first and last consultation in KUHR and/or NPR. If the patients had only one consultation, this was considered as both the first and the last. In addition, MSD overall included patients with shoulder/arm pain, joint, bone and cartilage disorders, soft tissue disorders and other MSD diagnoses (ICPC-2 and ICD-10 codes in Supplementary Table 1).

We used information about age and sex from the first consultation in KUHR. The types of healthcare professionals included GP and PT from KUHR, and all medical specialists in SHC from NPR. Information on consultation dates, reimbursement rate, and diagnosis code from both KUHR and NPR was used. From the individualized baseline consultation (could occur on any date in 2015), the number of MSD diagnoses, registered on each patient in KUHR, was counted. The total number of MSD diagnoses was extracted from baseline and over the subsequent two years, in both KUHR and NPR.

To identify trajectories based on healthcare use, the number of GP, PT and SHC consultations were extracted for four time periods during follow-up: 0–6, >6–12, >12–18 and >18–24 months from the first consultation.

Education was categorized into four categories (elementary school, high school (includes tertiary vocational school), university ≤4 years, university >4 years). Country of origin is defined by SSB as the country where patients were born, or where their mother was born for patients born in Norway with foreign parents: Norway, Western countries (other Western European countries, North America, Oceania), Eastern Europe, Africa, Asia and South America [30]. Due to low number of patients, university ≤4 and >4 years were collapsed into university and Africa, Asia and South America were collapsed into *Other* in the statistical analysis.

### Statistical analysis

Patient characteristics are presented as frequencies (%) and means (standard deviations, SDs) as appropriate.

To identify subgroups of patients with similar patterns of healthcare use over the two-year period, we used group-based multi-trajectory modelling described by Nagin et al. (2018) [31]. This method allows for the identification of treatment trajectory classes for multiple outcomes. We used the number of GP, PT and SHC consultations within the four time periods in our analysis. To identify the most appropriate number of classes, we tested models with one to six classes using time since baseline as time indicator, with quadratic terms and a zero-inflated Poisson model, for all MSD patients, as well as for each of the diagnostic groups (spine pain, FM and OA). Selection of the optimal number of classes was based on (1) goodness-of-fit criteria: Akaike Information Criterion (AIC) and Bayesian Information Criterion (BIC); (2) ≈5% of the patients per class; (3) mean predicted posterior probability of class membership above 70% in each class; and (4) the clinical validity of the treatment trajectory classes. Supplementary Table 2 provides information about this process. To ensure the convergence of our models, we truncated high numbers of PT consultations and restricted these to the 95th percentile.

Multinomial logistic regression was used to investigate whether education and country of origin were associated with treatment trajectory classes of healthcare use. To take the uncertainty of treatment trajectory classes into account, each patient was duplicated and assigned to each of the classes with its relative posterior probability of the class membership from the trajectory analysis. Then the posterior probabilities were used as weights in the regression analyses. Based on directed acyclic graphs we adjusted for age, sex, and country of origin in the analysis of education (Supplementary Figure 1), while no adjustment was needed in the analysis of country of origin (Supplementary Figure 2). Predicted probabilities with 95% confidence intervals (CIs) for being assigned to each trajectory class, by education and country of origin, were also calculated and presented in forest plots, and odds ratios (ORs) with 95% CIs in supplementary materials. A chi-square test was used to test the overall associations between the treatment trajectory classes and education and country of origin.

We used Stata version 17 for the statistical analyses, the traj package to create treatment trajectory classes, mlogit for multinomial logistic regression [32] and R version 4.1.3 for the forest plots [33]. A 5% level of significance was used.

## Results

### Study population

**Figure 1.**
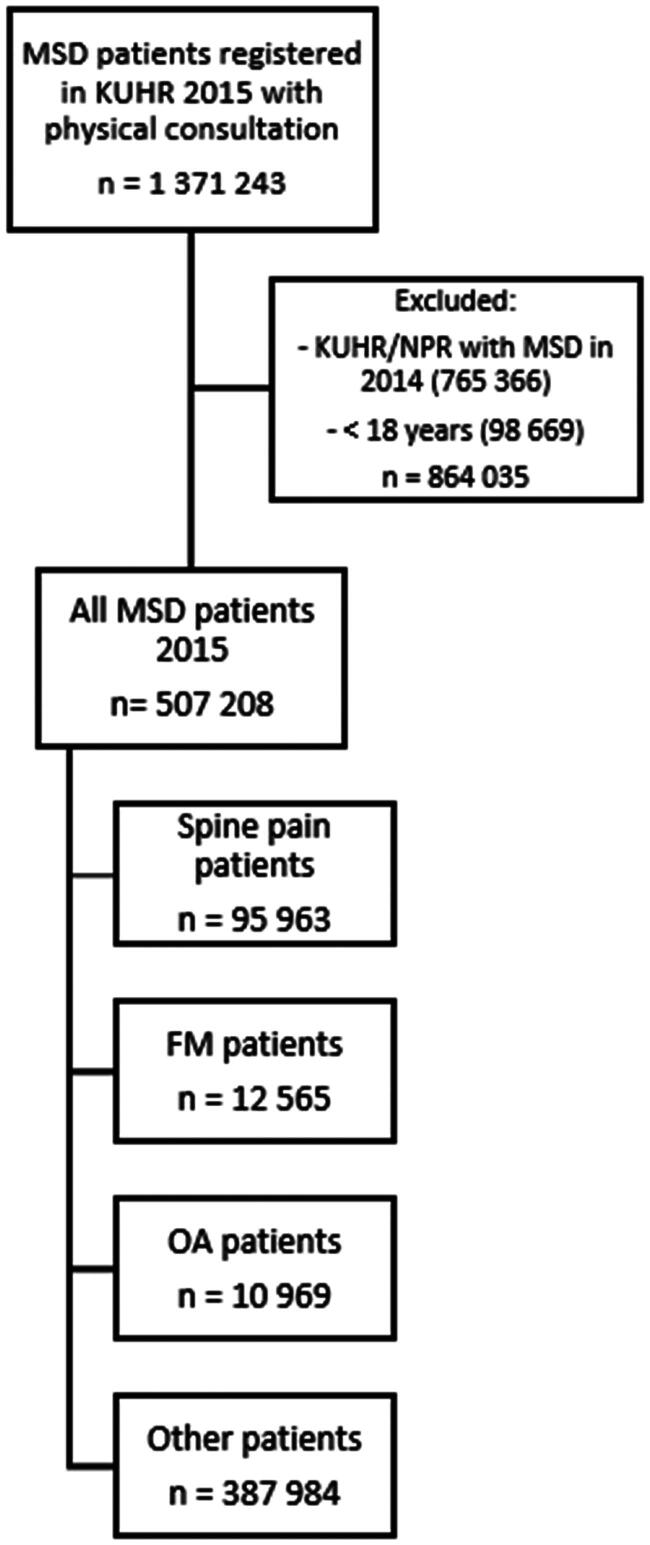
Flow diagram for inclusion of patients with musculoskeletal disorders (MSDs) registered in the Norwegian control and payment of health reimbursements database (KUHR; primary health care) in 2015. NPR = Norwegian Patient Registry; specialist health care, FM = fibromyalgia, OA = osteoarthritis.

In 2015, 1 371 243 patients were registered in KUHR with a physical consultation and at least one L-diagnosis (Figure 1). We excluded patients registered with MSD diagnoses in KUHR and/or NPR during the 12 months prior to the first contact in 2015 (*n* = 765 366) and patients <18 years (*n* = 98 699), resulting in a study sample of 507 208 patients with MSDs. There were 95 963 patients with spine pain, 12 565 with FM and 10 969 with OA.

### Characteristics

The mean (SD) age of the 507 208 patients with MSD was 48 (18) years and 51% were women (Table 1). Most patients had Norway as country of origin (78%) followed by Western countries (11%). Almost one-third had university education. At the baseline consultation, 93% were registered with only one MSD diagnosis, while 45% received ≥2 during the two-year follow-up period. The spine pain patients had the lowest proportion with Norway as country of origin (72%) and the lowest proportion with ≥2 MSDs at baseline (4%) compared to FM and OA patients. Patients with FM had the largest proportion of women (67%) and differed on the fraction with ≥2 MSDs at baseline (7%) compared to spine pain (4%) and OA patients (5%). Patients with OA were oldest (mean 67 years), had the highest proportion with Norway as country of origin (90%) and the lowest proportion with >4 years of university education.

**Table 1.** Characteristics and diagnostic information for patients with musculoskeletal disorders (MSDs) overall (*n* = 507 208) and in the three diagnostic groups (spine pain, fibromyalgia and osteoarthritis).

	All MSD	Spine pain	Fibromyalgia	Osteoarthritis
Number of patients	507 208	95 963	12 565	10 696
Age, mean (SD^1^)	48.1 (17.8)	44.8 (16.9)	44.9 (16.3)	67.4 (12.7)
Women, n (%)	259 540 (51.1)	48 279 (50.3)	8 473 (67.4)	6 379 (59.6)
Country of origin, n (%)				
Norway	398 033 (78.5)	69 303 (72.2)	9 511 (75.7)	9 571 (89.5)
Western countries	54 044 (10.7)	11 454 (12.0)	1 309 (10.4)	705 (6.6)
Eastern Europe	16 144 (3.2)	4 796 (5.0)	367 (2.9)	98 (0.9)
Africa	10 562 (2.1)	2 838 (3.0)	389 (3.1)	60 (0.6)
Asia	24 652 (4.8)	6 490 (6.7)	872 (7.0)	228 (2.1)
South America	3 059 (0.6)	656 (0.7)	73 (0.6)	22 (0.2)
Missing	714 (0.1)	426 (0.4)	44 (0.3)	11 (0.1)
Education, n (%)				
Elementary school	120 106 (23.7)	23 319 (24.3)	3 306 (27.0)	2 769 (25.9)
High school	218 917 (43.2)	39 187 (40.8)	5 090 (41.6)	5 147 (48.1)
University ≤ 4 years	115 976 (22.9)	22 036 (23.0)	2 898 (23.7)	2 008 (18.8)
University > 4 years	39 314 (7.7)	7 772 (8.1)	880 (7.2)	613 (5.7)
Missing	12 895 (2.5)	3 649 (3.8)	391 (3.1)	159 (1.5)
≥2 MSDs at baseline, n (%)	35 981 (7.1)	3 746 (3.9)	820 (6.5)	544 (5.3)
≥2 MSDs during follow-up, n (%)	226 212 (44.5)	21 403 (22.3)	2 790 (22.2)	4 771 (44.6)

### Treatment trajectory classes

**Figure 2.**
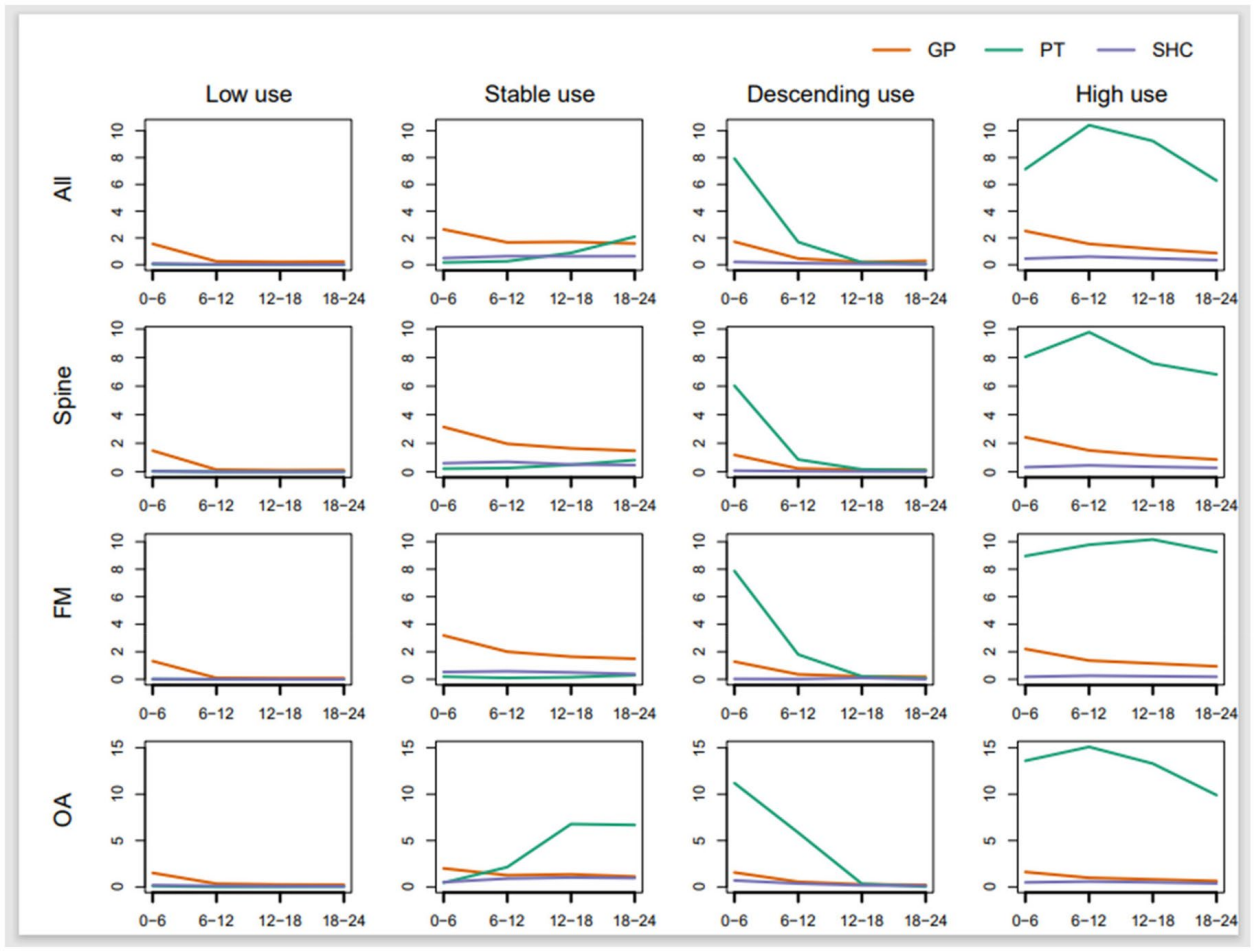
Average predicted number of general practitioner (GP) consultations, physiotherapy (PT) consultations and specialist health care (SHC) consultations in the four identified treatment trajectory classes of health care use (low, stable, descending and high use) during 24 months for musculoskeletal disorder (MSD) patients in Norway overall and in the three diagnostic groups (spine pain, fibromyalgia (FM) and osteoarthritis (OA)). Time since diagnosis (in months) is presented on the x-axis and average predicted number of consultations on the y-axis. Note that the y-axis is different for the patients with OA.

Using group-based multi-trajectory modelling and the selection criteria of optimal number of classes, we identified four treatment trajectory classes of healthcare use during the two-year follow-up period among MSD patients overall and in the three diagnostic groups. After visual inspecting the average predicted trajectories for the three types of health providers (GP, PT and SHC), we labelled the four treatment trajectory classes low, stable, descending and high use (Figure 2). About 75% of all the MSD patients were classified with low use, 10%, 9% and 5% with stable, descending, and high use, respectively (Table 2). The patients categorized as low users consulted mainly the GP and had about two consultations within the first 6 months, and no further consultations thereafter (Figure 2). GP consultations also dominated among the stable users, except for patients with OA who consulted mainly PTs 12 to 24 months after baseline. The trajectory class with descending healthcare use had a high number of PT consultations within the first 6 months, reduced to almost none after one year. High users had fairly constant use of consultations with all healthcare professionals during the two-year follow-up, including high use of PT consultations.

**Table 2.** Characteristics, diagnostic information and number of consultations by treatment trajectory classes of health care use for patients with musculoskeletal disorders (MSDs) overall (*n* = 507 208) and in the three diagnostic groups (spine pain, fibromyalgia, osteoarthritis).

	Treatment trajectory classes of health care use			
	Low use	Stable use	Descending use	High use
***All MSD (n = 507 208)***				
Number of patients, n (%)	382 010 (75.3)	51 343 (10.3)	46 785 (9.3)	26 365 (5.2)
Age, mean (SD^1^]	47.1 (18.0)	49.8 (16.5)	50.1 (17.4)	54.7 (16.1)
Women, n (%)	188 478 (49.1)	28 504 (44.5)	26 512 (56.6)	16 045 (60.9)
≥2 MSDs at baseline, n (%)	28 952 (7.5)	6 629 (7.7)	2 119 (4.5)	930 (3.5)
≥2 MSDs during follow-up, n (%)	133 240 (34.7)	45 260 (88.1)	25 503 (54.4)	22 209 (84.2)
Consultations, mean (SD)				
Total	2.4 (1.8)	13.6 (7.7)	13.6 (9.2)	47.1 (28.1)
GP [2]	2.2 (1.6)	7.7 (5.1)	2.8 (2.9)	6.1 (5.1)
PT [3]	0.1 (0.3)	3.6 (5.6)	10.3 (8.3)	39.4 (26.4)
SHC [4]	0.2 (0.6)	1.8 (3.0)	0.5 (1.1)	1.9 (3.0)
***Spine pain (n = 95 963)***				
Number of patients, n (%)	69 390 (72.6)	10 058 (10.5)	11 234 (11.8)	4 855 (5.1)
Age, mean (SD)	44,3 (17.1)	45,3 (15.3)	45.1 (16.8)	49,2 (15.8)
Women, n (%)	33 784 (48.4)	4 901 (48.7)	6 583 (58.5)	3 011 (62.0)
≥2 MSDs at baseline, n (%)	2 932 (4.2)	533 (5.3)	173 (1.5)	108 (2.2)
≥2 MSDs during follow-up, n (%)	8560 (12.3)	7 507 (74.6)	2 362 (21.0)	2 975 (61.2)
Consultations, mean (SD)				
Total	2.1 (1.4)	12.3 (7.2)	9.1 (6.6)	42.5 (25.5)
GP	1.9 (1.4)	8.2 (5.1)	1.7 (2.3)	5.9 (5.7)
PT	0 (0.2)	1.8 (3.0)	7.3 (5.8)	35.2 (24.8)
SHC	0.2 (0.3)	2.3 (4.2)	0.2 (0.6)	1.4 (3.1)
***Fibromyalgia (n = 12 565)***				
Number of patients	9 837 (78.6)	1 256 (10.0)	912 (7.3)	516 (4.1)
Age, mean (SD)	44.3 (16.7)	46,6 (13.1)	47.3 (16.3)	47.9 (13.8)
Women, n (%)	6 291 (63.7)	1 038 (82.6)	707 (77.4)	437 (84.7)
≥2 MSDs at baseline, n (%)	669 (6.8)	116 (9.2)	21 (2.3)	14 (2.7)
≥2 MSDs during follow-up, n (%)	1 245 (12.6)	736 (76.0)	69 (29.0)	246 (63.0)
Consultations, mean (SD)				
Total	1.6 (1.1)	11.1 (7.3)	12.1 (7.7)	44.5 (25.4)
GP	1.6 (1.1)	8.3 (5.3)	2.0 (2.5)	5.5 (5.4)
PT	0 (0.2)	0.7 (1.9)	10.0 (7.2)	38.1 (25.1)
SHC	0 (0.2)	2.0 (4.6)	0.1 (0.3)	0.8 (2.3)
***Osteoarthritis (n = 10 696)***				
Number of patients	7 065 (66.0)	907 (8.5)	1 578 (14.8)	1 146 (10.7)
Age, mean (SD)	67.4 (13.5)	67.0 (10.5)	68.0 (11.6)	67.5 (10.3)
Women, n (%)	4 137 (58.6)	557 (61.4)	951 (60.3)	734 (64.1)
≥2 MSDs at baseline, n (%)	480 (7.1)	25 (2.8)	24 (1.5)	13 (1.2)
≥2 MSDs during follow-up, n (%)	2 115 (30.1)	748 (87.1)	908 (58.3)	852 (75.0)
Consultations, mean (SD)				
Total	2.9 (2.7)	27.7 (15.8)	23.0 (14.1)	72.0 (34.4)
GP	2.4 (2.2)	5.7 (4.5)	2.6 (2.7)	4.0 (3.5)
PT	0.1 (0.6)	18.5 (14.7)	19.0 (13.4)	66.0 (34.1)
SHC	0.5 (1.1)	3.4 (4.3)	1.34 (2.0)	2.0 (2.5)

^1^SD = Standard deviation.

^2^GP = General practitioner.

^3^PT = Physiotherapy.

^4^SHC = Specialist health care.

#### MSD patients overall

Among MSD patients overall, the low users were the youngest (mean 47 years) and balanced as regards to sex. At baseline, 8% were registered with ≥2 MSDs and the fraction increased to 35% during follow-up (Table 2). Stable users were slightly older (mean 50 years), more were men (56%), at baseline 8% had ≥2 MSDs increasing to 88% during follow-up. Mean age of the descending users was also 50 years, 57% were women, 5% had ≥2 MSDs at baseline and increased to 54% during follow-up. The high users were the oldest (mean 55 years), had the highest proportion of women (61%) and almost all (96%) had only one MSD at baseline while 84% had ≥2 MSDs during follow-up. The mean number of consultations was much higher among high (47.1) compared to low users (2.4), with PT consultations as the main contributor among high users. Stable and descending users had a similar number of total consultations (both 13.6), but the distribution between GP, PT and SHC differed.

#### Spine pain

The characteristics of spine pain patients within the treatment trajectory classes were similar to the MSD patients overall, with a slightly higher proportion of descending users and lower proportion of PT consultations within the treatment trajectory classes (Table 2).

#### Fibromyalgia (FM)

Among patients with FM, a higher proportion were low users compared to both the MSD patients overall and the other two diagnostic groups. Although the proportion of women was high in all the treatment trajectory classes with FM, the proportion of men among low users was relatively higher than among spine pain and OA patients.

#### Osteoarthritis (OA)

The characteristics of patients with OA differed the most from the MSD patients overall and from the other diagnostic groups. The total number of consultations were higher in all the treatment trajectory classes and less patients were low and stable users. The mean age was similar within the treatment trajectory classes, in contrast to the other groups.

### Associations between the treatment trajectory classes and education and country of origin

**Figure 3.**
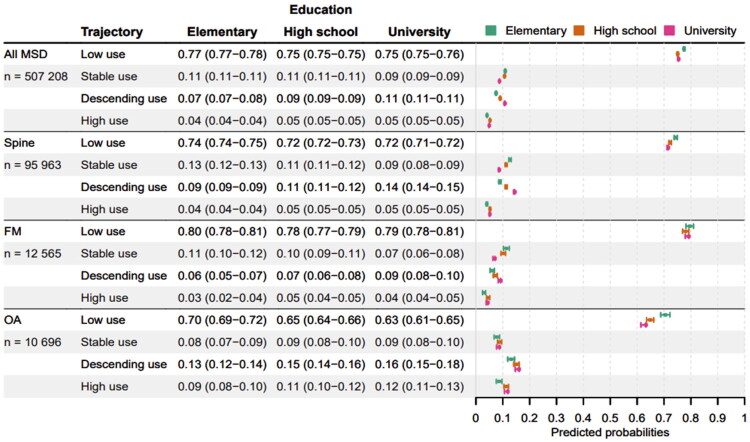
Forrest plot showing the predicted probabilities of being assigned to each treatment trajectory classes of health care use (low, stable, descending and high use) with 95% confidence intervals, by education, for all MSD patients and in the three diagnostic groups (spine pain, fibromyalgia (FM) and osteoarthritis (OA)), respectively. Probabilities were calculated from multinomial logistic regression adjusted for age, sex and country of origin.

Education was significantly associated with the treatment trajectory classes of healthcare use for MSD patients overall and for the diagnostic groups (*p* < 0.01; Supplementary Table 3). Among MSD patients overall, the probability of being a low user decreased slightly with increasing education (77% for elementary school and 75% for university education). Similarly, the probability of being a stable user decreased with increasing education (11% for elementary school and 9% for university education), while the probability of being a descending user increased with higher education (7% for elementary and 11% for university education) (Figure 3). The probabilities of being high users were almost similar across education levels among the MSD patients overall (4–5%).

The association between education and the treatment trajectory classes was similar among patients with spine pain, FM and MSD patients overall, except for the low users with FM where no association with education was identified (Figure 3). Among OA patients, those with elementary school were more likely to be low users (70%) compared to those with high school and university education (63-65%).

**Figure 4.**
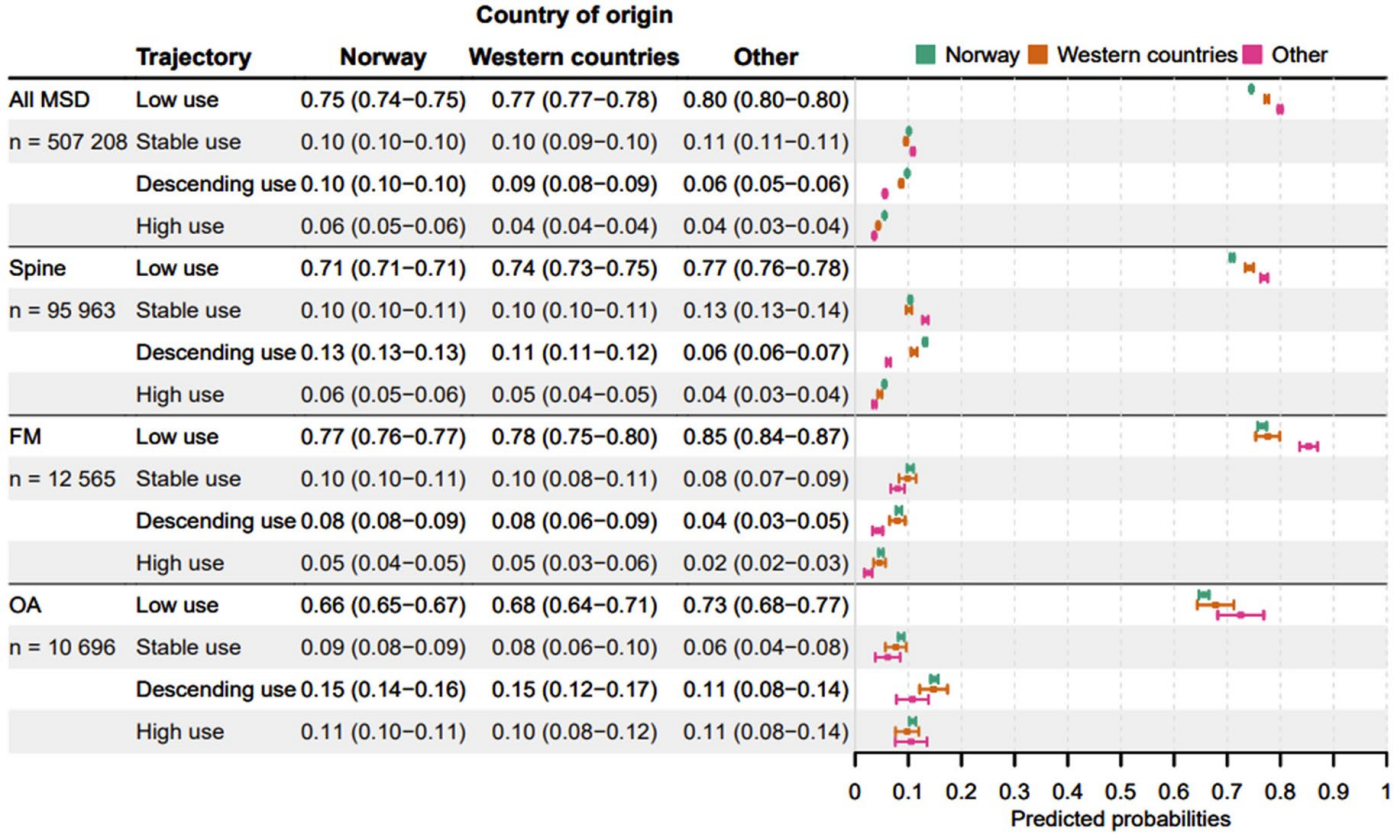
Forrest plot showing the predicted probabilities of being assigned to each treatment trajectory classes of health care use (low, stable, descending and high use) with 95% confidence intervals by country of origin, for all MSD patients and by diagnostic groups (spine pain, fibromyalgia (FM) and osteoarthritis (OA)), respectively. Probabilities were calculated from multinomial logistic regression.

Country of origin was significantly associated with treatment trajectory classes of healthcare use in MSD patients overall and in all the diagnostic groups (*p* < 0.002; Supplementary Table 4). Among MSD patients overall, those with Western and *Other* country of origin had higher probabilities of being low users (77-80% versus 75% for those with Norway as country of origin) (Figure 4). Patients with origin from Norway and Western countries had higher probabilities of being descending users compared to patients with other country of origin (9–10% vs. 6%). The probabilities of being stable and high users were almost similar across country of origin (10–11% and 4–6%, respectively).

The association between country of origin and treatment trajectory classes was similar among spine pain patients and MSD patients overall, except for stable users. Spine patients with *Other* country of origin had higher probabilities of being stable users than those with Norway and Western countries of origin (13% and 10%, respectively). FM patients with other country of origin were more likely to be low users (85% vs. 77–78%) compared to FM patients with Norway and Western countries of origin, and less likely to be descending users (4% vs. 8%). Among OA patients, the probability of being low user was higher among patients with other country of origin than Norway (73% and 66%, respectively) (Figure 4).

## Discussion

### Summary of findings

Using group-based multi-trajectory modelling, we identified four treatment trajectory classes of healthcare use among patients with MSD over a two-year period: low, stable, descending, and high use. About 75% of the MSD patients overall were low users, which corresponds to having approximately two GP consultations during the first 6 months. Ten percent were stable users with an even distribution of healthcare use throughout the two-year follow-up. Nine percent were descending users with high healthcare use within the first 12 months, followed by a decline in the second year. Finally, the high users (5%) had consistent high healthcare use, especially PT services, during the whole period. The treatment trajectory classes of the three diagnostic groups were quite similar as for all MSD patients. Low education increased the probability of being a stable user, while higher education increased the probability of being a descending user. Patients with *Other* country of origin were more prone to be low users, while patients with origin from Norway and Western countries had higher probability of being descending users compared to patients with other country of origins.

### Treatment trajectory classes

#### Between classes

In our study, most patients diagnosed with MSDs were low users of healthcare services with only 5% high users over the two-year follow-up. Previous studies have also found a small percentage of MSD patients with significantly more healthcare use than others [3–5]. We also identified treatment trajectory classes of stable and descending users with a similar number of consultations, but the distribution between GP, PT and SHC differed. Previous studies have found that SHC services are more expensive than PHC, and that PT services are more cost-effective than GP and SHC services [34–36]. Hence, our stable users might have been considered as high users, if we had performed health economy analyses, since they had high use of SHC services.

The substantial contrast in the distribution of sex between the treatment trajectory classes in our study is notable. Women are previously found to be more prone to develop long-lasting MSDs than men [9,37], and more frequent users of healthcare services, especially PT services [38]. These factors may contribute to the higher proportion of women among both the descending and high users in our study. Another interesting result is the majority of younger men among the stable users. Previous studies have found lower health literacy among men compared to women [39]. This may contribute to the understanding of the prolonged use of treatment [40]. More young men among the stable users may also be due to the increasing prevalence of mental illness among this group [41] since mental illness is associated with long-lasting MSD [42]. Unfortunately, we had no information in our data to explore this further.

#### Diagnostic groups

The distribution of patients across the four treatment trajectory classes differed slightly between the diagnostic groups. FM patients had the highest proportion of low users. This is surprising, since FM is considered a long-lasting chronic disorder and is seen more as a diagnosis of exclusion [43], hence higher use can be expected. On the other hand, the treatment options for FM are limited, and follow-up is recommended to be in PHC for these patients. A referral to SHC should be considered only when there is suspicion of other conditions [43]. The low number of consultations in SHC in our study for FM patients may illuminate this. Moreover, patient education, cognitive therapy and physical activity with alternatively pharmacological intervention are treatment recommendations all handled in the PHC^44^. However, due to widespread pain and fatigue, physical activity can be challenging [45]. PT treatment is often associated with physical activity and may explain the low proportion of descending and high users among patients with FM.

The higher proportion of descending and high users among OA patients compared to the MSD patients overall and the two diagnostic groups may be due to the large use of PT services. This can be seen as being in accordance with clinical guidelines, recommending exercise therapy for OA patients [46]. However, a natural endpoint for these patients is joint replacements, and this can be reflected in the relatively high number of SHC consultations found in all classes.

### Education

We found that low education increased the probability of being both a low and a stable user of healthcare services in the MSD patients overall as well as for spine pain patients. Low education has previously been associated with lower health literacy and limited financial resources [47,48] and may explain the low use, especially within the first 6 months, compared to the descending and high users of healthcare services. Further, we found that higher education increased the probability of being a descending user. Descending use might indicate a more efficient use of healthcare services and might reflect faster recovery. High education has been associated with a healthier and more active lifestyle, higher health literacy and better financial resources which are important components in recovery from MSDs [49]. However, several of these factors, health literacy, lifestyle factors, and healthcare access, are unknown in our register study and should be addressed in future studies. Surprisingly, the patients with FM neither differed in educational level from the general population nor from the MSD patients overall. This is in contrast to previous findings where FM was associated with low education [50].

### Country of origin

To our knowledge, no previous studies have investigated and compared healthcare use among MSD patients with different country of origin living in Norway. Previous research has shown that immigrants in Norway have reported a higher prevalence of MSDs compared to native Norwegians, explained by work-related and psychosocial factors related to migration [30]. In line with this, the proportion of MSD patients with a country of origin other than Norway in our study cohort was higher than the proportion of immigrants in the general population [51]. At the same time, we found that patients with Western and *Other* country of origin were more likely to be low users and less likely to be descending users of healthcare services compared to patients with Norway as country of origin. This is consistent with earlier findings showing that immigrants use healthcare services less frequently than natives, particularly the PT services [22] and have less referrals to SHC [52]. These factors may have influenced the distribution of immigrants in the treatment trajectory classes in our study. Our findings highlight the importance of examining healthcare use across groups with different countries of origin to ensure equitable healthcare services. Cultural aspects, language barriers, health literacy, and financial resources have been suggested as possible causes for these differences [53].

### Strengths and limitations

The extensive coverage of all patients consulting the publicly funded healthcare services in Norway is an important strength of this register study. Comprehensive data secured minimal selection bias, no recall bias and allowed for robust statistical analyses. This enhances the generalizability of the results for the entire population. The lack of information about patients treated in private clinics should be taken into account. About 10% of healthcare services in Norway were provided in private clinics during the study period, either fully paid by private insurance companies or by the patients themselves [14]. Chiropractor consultations registered in KUHR were not included in the present study due to low numbers. This may represent a limitation, especially for the analyses involving the spine pain patients. Information about socioeconomic status, which is often based on a combination of education and income, was not requested and might be seen as a limitation. Moreover, healthcare services provided in nursing homes are not included in the registers, and this may have influenced the age distribution in the study.

The ability to link data from different registers, using the unique Norwegian national identification number, enabled us to follow patients over time, both in primary and specialist healthcare. The linkage to Statistics Norway provided information about education and country of origin for every patient.

MSDs often have long-lasting or recurrent nature, and most of these conditions do not require continuous treatment. A longer ‘washout’ period than one year might have given an even more precise selection of patients with a new episode of MSD.

The validity of diagnostic coding (ICPC-2 and ICD-10), used to identify all patients with MSD and the three diagnostic groups, has been questioned, especially in primary healthcare. However, a previous Norwegian study found an adequate association between ICPC diagnoses and patient record notes [54]. The codes have also been considered as a reliable classification system for comparing groups across primary and specialist healthcare [55].

## Conclusion

Our results from using nationwide register data and group-based multi-trajectory modelling show that about 75% of patients with MSDs were classified as low users of healthcare services within two-years follow-up, while 5% were high users. The treatment trajectory class with low use was associated with another country of origin than Norway. Descending users (high initial use followed by a decline after the first year) was associated with high education and Norwegian and Western country of origin. High use was neither associated with education nor country of origin. Patients with OA were more likely be high users of healthcare services compared to patients with MSD overall and spine pain and FM. Hence, our result highlights the important of sociodemographic and diagnostic differences in healthcare use patterns.

## Supplementary Material

Supplementary_revised_28_January.docx

Supplementary_Article_2_june.docx

## References

[1] Dieleman JL, Cao J, Chapin A, et al. US health care spending by payer and health condition, 1996-2016. JAMA. 2020;323(9):863–884. doi: 10.1001/jama.2020.0734. 32125402 PMC7054840

[2] Cieza A, Causey K, Kamenov K, et al. Global estimates of the need for rehabilitation based on the global burden of disease study 2019: a systematic analysis for the global burden of disease study 2019. Lancet. 2021;396(10267):2006–2017. doi: 10.1016/S0140-6736(20)32340-0. 33275908 PMC7811204

[3] Lentz TA, Harman JS, Marlow NM, et al. Factors associated with persistently high-cost health care utilization for musculoskeletal pain. PLoS One. 2019;14(11):e0225125. doi: 10.1371/journal.pone.0225125. 31710655 PMC6844454

[4] Mose S, Kent P, Smith A, et al. Trajectories of musculoskeletal healthcare utilization of people with chronic musculoskeletal pain - a population-based cohort study. Clin Epidemiol. 2021;13:825–843. doi: 10.2147/CLEP.S323903. 34557040 PMC8455515

[5] Amundsen O, Moger TA, Holte JH, et al. Combination of health care service use and the relation to demographic and socioeconomic factors for patients with musculoskeletal disorders: a descriptive cohort study. BMC Health Serv Res. 2023;23(1):858. 20230814. doi: 10.1186/s12913-023-09852-3. 37580723 PMC10426198

[6] Rosella LC, Fitzpatrick T, Wodchis WP, et al. High-cost health care users in Ontario, Canada: demographic, socio-economic, and health status characteristics. BMC Health Serv Res. 2014;14(1):532. 20141031. doi: 10.1186/s12913-014-0532-2. 25359294 PMC4221677

[7] Buchbinder R, van Tulder M, Oberg B, et al. Low back pain: a call for action. Lancet. 2018;391(10137):2384–2388. 20180321 doi: 10.1016/S0140-6736(18)30488-4. 29573871

[8] Ihlebaek C, Laerum E. [Hits most, costs most and gets least]. Tidsskr nor Laegeforen. 2010;130(21):2106. doi: 10.4045/tidsskr.10.1035. 21052101

[9] Kinge JM, Knudsen AK, Skirbekk V, et al. Musculoskeletal disorders in Norway: prevalence of chronicity and use of primary and specialist health care services. BMC Musculoskelet Disord. 2015;16(1):75–20150402. doi: 10.1186/s12891-015-0536-z. 25887763 PMC4392859

[10] Fitzmaurice C, Allen C, Global Burden of Disease Cancer C., et al. Global, regional, and national cancer incidence, mortality, years of life lost, years lived with disability, and disability-adjusted life-years for 32 cancer groups, 1990 to 2015: a systematic analysis for the global burden of disease study. JAMA Oncol. 2017;3:524–548. doi: 10.1001/jamaoncol.2016.5688. 27918777 PMC6103527

[11] Marques AP, Santo A, Berssaneti AA, et al. Prevalence of fibromyalgia: literature review update. Rev Bras Reumatol Engl Ed. 2017;57(4):356–363. 20170208 doi: 10.1016/j.rbre.2017.01.005. 28743363

[12] Benebo FO, Lukic M, Jakobsen MD, et al. Lifestyle risk factors of self-reported fibromyalgia in the Norwegian women and cancer (NOWAC) study. BMC Public Health. 2023;23(1):1967. 20231011. doi: 10.1186/s12889-023-16773-7. 37821848 PMC10566054

[13] Hallberg S, Rolfson O, Karppinen J, et al. Burden of disease and management of osteoarthritis and chronic low back pain: healthcare utilization and sick leave in Sweden, Norway, Finland and Denmark (BISCUITS): study design and patient characteristics of a real world data study. Scand J Pain. 2023;23(1):126–138. 20220720 doi: 10.1515/sjpain-2021-0212. 35858277

[14] Saunes IS, Karanikolos M, Sagan A. Norway: health system review. Health Syst Transit. 2020;22(1):1–163. 32863241

[15] Oliveira CB, Maher CG, Pinto RZ, et al. Clinical practice guidelines for the management of non-specific low back pain in primary care: an updated overview. Eur Spine J. 2018;27(11):2791–2803. 20180703 doi: 10.1007/s00586-018-5673-2. 29971708

[16] Tyrdal MK, Perrier F, Røe C, et al. Musculoskeletal disorders in Norway: trends in health care utilization and patient pathways: a nationwide register study. Scand J Prim Health Care. 2024;42(4):582–592. 20240721. doi: 10.1080/02813432.2024.2368848. 39034654 PMC11552292

[17] Lacasse A, Nguena Nguefack HL, Page G, et al. Sex and gender differences in healthcare utilisation trajectories: a cohort study among Quebec workers living with chronic pain. BMJ Open. 2023;13(7):e070509. 20230730. doi: 10.1136/bmjopen-2022-070509. PMC10387645 37518085

[18] Nguena Nguefack HL, Pagé MG, Choinière M, et al. Distinct care trajectories among persons living with arthritic conditions: a two-year state sequence analysis. Front Pain Res. 2022;3(01):14. doi: 10.3389/fpain.2022.1014793. PMC9699830 36444387

[19] Ruetsch C, Tkacz J, Kardel PG, et al. Trajectories of health care service utilization and differences in patient characteristics among adults with specific chronic pain: analysis of health plan member claims. J Pain Res. 2013;6:137–149. 20130221 doi: 10.2147/jpr.S38301. 23459176 PMC3583440

[20] Hansen KL, Melhus M, Lund E. Ethnicity, self-reported health, discrimination and socio-economic status: a study of Sami and non-Sami Norwegian populations. Int J Circumpolar Health. 2010;69(2):111–128. 20100401 doi: 10.3402/ijch.v69i2.17438. 20359443

[21] Finnvold JE. How social and geographical backgrounds affect hospital admission with a serious condition: a comparison of 11 immigrant groups with native-born Norwegians. BMC Health Serv Res. 2018;18(1):843. 20181108. doi: 10.1186/s12913-018-3670-0. 30409144 PMC6225619

[22] Diaz E, Gimeno-Feliu LA, Calderón-Larrañaga A, et al. Frequent attenders in general practice and immigrant status in Norway: a nationwide cross-sectional study. Scand J Prim Health Care. 2014;32(4):232–240. 20141125 doi: 10.3109/02813432.2014.982368. 25421090 PMC4278396

[23] Ruud SE, Hjortdahl P, Natvig B. Is it a matter of urgency? A survey of assessments by walk-in patients and doctors of the urgency level of their encounters at a general emergency outpatient clinic in Oslo, Norway. BMC Emerg Med. 2016;16(1):22. 20160704. doi: 10.1186/s12873-016-0086-1. 27378228 PMC4932670

[24] Corscadden L, Levesque JF, Lewis V, et al. Factors associated with multiple barriers to access to primary care: an international analysis. Int J Equity Health. 2018;17(1):28. 20180220. doi: 10.1186/s12939-018-0740-1. 29458379 PMC5819269

[25] Berkman ND, Sheridan SL, Donahue KE, et al. Low health literacy and health outcomes: an updated systematic review. Ann Intern Med. 2011;155(2):97–107. doi: 10.7326/0003-4819-155-2-201107190-00005. 21768583

[26] Czapka EA, Sagbakken M. “Where to find those doctors?” A qualitative study on barriers and facilitators in access to and utilization of health care services by Polish migrants in Norway. BMC Health Serv Res. 2016;16(1):460. 20160901. doi: 10.1186/s12913-016-1715-9. 27586150 PMC5007991

[27] Helsedata.no. Data sources: KUHR and NPR. https://helsedata.no/en/data-sources/?page=1&sort=0 (2024).

[28] Health Do. KUHR-databasen. https://www.helsedirektoratet.no/tema/statistikk-registre-og-rapporter/helsedata-og-helseregistre/kuhr. (2019).

[29] Health Do. Finn kode. https://finnkode.helsedirektoratet.no/#icpc/0/0/0/2854541 (2024).

[30] Kjøllesdal MKR, Gerwing J, Indseth T. Health risks among long-term immigrants in Norway with poor Norwegian language proficiency. Scand J Public Health. 2023;51(3):422–429. doi: 10.1177/14034948221085399. 35548943

[31] Nagin DS, Jones BL, Passos VL, et al. Group-based multi-trajectory modeling. Stat Methods Med Res. 2018;27(7):2015–2023. 20161017 doi: 10.1177/0962280216673085. 29846144

[32] Stata Statistical Software 18.2023

[33] R: a language and environment for statistical computing. Foundation for Statistical Computing. 2021. DOI: https://www.R-project.org

[34] Amundsen O, Moger TA, Holte JH, et al. Patient characteristics and healthcare use for high-cost patients with musculoskeletal disorders in Norway: a cohort study. BMC Health Serv Res. 2024;24(1):1583. 20241218. doi: 10.1186/s12913-024-12051-3. 39695620 PMC11653887

[35] Bornhöft L, Thorn J, Svensson M, et al. More cost-effective management of patients with musculoskeletal disorders in primary care after direct triaging to physiotherapists for initial assessment compared to initial general practitioner assessment. BMC Musculoskelet Disord. 2019;20(1):186. 20190501. doi: 10.1186/s12891-019-2553-9. 31043169 PMC6495522

[36] Piscitelli D, Furmanek MP, Meroni R, et al. Direct access in physical therapy: a systematic review. Clin Ter. 2018;169(5):e249–e260. doi: 10.7417/ct.2018.2087. 30393813

[37] Wijnhoven HA, de Vet HC, Picavet HS. Prevalence of musculoskeletal disorders is systematically higher in women than in men. Clin J Pain. 2006;22(8):717–724. doi: 10.1097/01.ajp.0000210912.95664.53. 16988568

[38] Suominen-Taipale AL, Martelin T, Koskinen S, et al. Gender differences in health care use among the elderly population in areas of Norway and Finland. A cross-sectional analysis based on the HUNT study and the FINRISK senior survey. BMC Health Serv Res. 2006;6(1):110. 20060904. doi: 10.1186/1472-6963-6-110. 16952306 PMC1569836

[39] Clouston SAP, Manganello JA, Richards M. A life course approach to health literacy: the role of gender, educational attainment and lifetime cognitive capability. Age Ageing. 2017;46(3):493–499. doi: 10.1093/ageing/afw229. 27940567 PMC5405758

[40] Shahid R, Shoker M, Chu LM, et al. Impact of low health literacy on patients’ health outcomes: a multicenter cohort study. BMC Health Serv Res. 2022;22(1):1148. doi: 10.1186/s12913-022-08527-9. 36096793 PMC9465902

[41] Sivertsen B, Knudsen AKS, Kirkøen B, et al. Prevalence of mental disorders among Norwegian college and university students: a population-based cross-sectional analysis. Lancet Reg Health Eur. 2023;34:100732. 20230919. doi: 10.1016/j.lanepe.2023.100732. 37927428 PMC10624983

[42] Garnæs KK, Mørkved S, Tønne T, et al. Mental health among patients with chronic musculoskeletal pain and its relation to number of pain sites and pain intensity, a cross-sectional study among primary health care patients. BMC Musculoskelet Disord. 2022;23(1):1115. 20221222. doi: 10.1186/s12891-022-06051-9. 36544130 PMC9773452

[43] Häuser W, Fitzcharles MA. Facts and myths pertaining to fibromyalgia. Dialogues Clin Neurosci. 2018;20(1):53–62. doi: 10.31887/DCNS.2018.20.1/whauser. 29946212 PMC6016048

[44] Macfarlane GJ, Kronisch C, Dean LE, et al. EULAR revised recommendations for the management of fibromyalgia. Ann Rheum Dis. 2017;76(2):318–328. 20160704 doi: 10.1136/annrheumdis-2016-209724. 27377815

[45] Lazaridou A, Paschali M, Schreiber K, et al. The association between daily physical exercise and pain among women with fibromyalgia: the moderating role of pain catastrophizing. Pain Rep. 2020;5(4):e832. 20200727. doi: 10.1097/pr9.0000000000000832. 32766468 PMC7390593

[46] Bannuru RR, Osani MC, Vaysbrot EE, et al. OARSI guidelines for the non-surgical management of knee, hip, and polyarticular osteoarthritis. Osteoarthritis Cartilage. 2019;27(11):1578–1589. 20190703 doi: 10.1016/j.joca.2019.06.011. 31278997

[47] Jansen T, Rademakers J, Waverijn G, et al. The role of health literacy in explaining the association between educational attainment and the use of out-of-hours primary care services in chronically ill people: a survey study. BMC Health Serv Res. 2018;18(1):394. 20180531. doi: 10.1186/s12913-018-3197-4. 29855365 PMC5984471

[48] Duijvestijn M, de Wit GA, van Gils PF, et al. Impact of physical activity on healthcare costs: a systematic review. BMC Health Serv Res. 2023;23(1):572. 20230603. doi: 10.1186/s12913-023-09556-8. 37268930 PMC10239135

[49] Raghupathi V, Raghupathi W. The influence of education on health: an empirical assessment of OECD countries for the period 1995–2015. Arch Public Health. 2020;78(1):20. doi: 10.1186/s13690-020-00402-5. 32280462 PMC7133023

[50] Macfarlane GJ, Norrie G, Atherton K, et al. The influence of socioeconomic status on the reporting of regional and widespread musculoskeletal pain: results from the 1958 British Birth Cohort Study. Ann Rheum Dis. 2009;68(10):1591–1595. 20081024 doi: 10.1136/ard.2008.093088. 18952642

[51] Statistics-Norway. Immigrants and Norwegian-born to immigrant parents [Innvandrere og norskfødte med innvandrerforeldre]. https://www.ssb.no/en/statbank/table/05182/ (2025).

[52] Sarría-Santamera A, Hijas-Gómez AI, Carmona R, et al. A systematic review of the use of health services by immigrants and native populations. Public Health Rev. 2016;37(1):28. doi: 10.1186/s40985-016-0042-3. 29450069 PMC5810113

[53] Debesay J, Arora S, Bergland A. 4. Migrants’ consumption of healthcare services in Norway. Inclusionary and exclusionary structures and practices. 2019.

[54] Sporaland GL, Mouland G, Bratland B, et al. General practitioners’ use of ICPC diagnoses and their correspondence with patient record notes. Tidsskr nor Laegeforen. 2019;139(15):20191014. doi: 10.4045/tidsskr.18.0440. 31642635

[55] Ohm E, Holvik K, Madsen C, et al. Incidence of injuries in Norway: linking primary and secondary care data. Scand J Public Health. 2020;48(3):323–330. 20190411 doi: 10.1177/1403494819838906. 30973061

