# Supplementary material for: Treatment trajectories among patients with musculoskeletal disorders in Norway – a register-based cohort study over 2 years
Source: Scand J Prim Health Care. 2026 Feb 25;44(1):2633751. doi: 10.1080/02813432.2026.2633751 (PMC12943818; doi:10.1080/02813432.2026.2633751)
Supplement: Supplementary_revised_28_January.docx [file IPRI_A_2633751_SM5597.docx]

**Supplementary Table 1.** Diagnostic groups according to International Classification of Primary Care, second edition (ICPC-2) and International Classification of Diseases 10^th^ revision (ICD-10).

|  | ICPC-2-  L-chapter | ICD-10  M-chapter |
| --- | --- | --- |
| Spine pain | 01-03, 83-86, | 40-54 |
| Fibromyalgia | 18 | 79.7 |
| Osteoarthritis | 89-91 | 15-19 |
| Shoulder/arm pain | 08,92 | 75 |
| Joint, bone and cartilage disorders | 05,07,09-13,15-17,20,70,88,94-95, 98-99 | 00-03,05-14,20-25,82-94 |
| Soft tissue disorders | 14,18-19,87,93 | 60-63,65-68,70-78 |
| Other | 04,26-29,71,73-82,96-97 | 30-36, 79 (-79.7), 95-96,99 |

**Supplementary Table 2.** Reported BIC and AIC and change in percentage when increasing number of classes in the analysis of treatment trajectories.

| ***No. of groups*** | ***BIC*** | ***BIC %*** | ***AIC AIC%*** | | ***Group size %*** | ***Entropy*** |
| --- | --- | --- | --- | --- | --- | --- |
|  |  |  | **All MSDs** | |  |  |
| 2 | -5 369 996 | -100% | -5 369 890 -100% | | 85,15 | 0.983 |
| 3 | -4 966 789 | 7.5 | -4 966 628 7.5 | | 77,16,7 | 0.966 |
| 4 | -4 645 434 | 6.5 | -4 645 127 6.5 | | 5,75,10,9 | 0.969 |
| 5 | -4 483 169 | 3.5 | -4 482 896 3.5 | | 11,5,71,9,4 | 0.951 |
| 6 | -4 360 387 | 2.7 | -4 360 058 2.7 | | 12,4,4,70,2,8 | 0.953 |
|  |  |  | **Spine pain** | |  |  |
| 2 | -900 339 | -100% | -900 249 -100% | | 86,14 | 0.980 |
| 3 | -829 397 | 7.9 | -829 259 7.9 | | 12,79,9 | 0.960 |
| 4 | -757 434 | 8.7 | -757 249 8.7 | | 11,73,11,5 | 0.940 |
| 5 | -733 964 | 3.1 | -733 732 3.1 | | 9,4,72,11,4 | 0.963 |
| 6 | -734 095 | 0 | -733 742 0 | | * | 1.000 |
|  |  |  | **Fibromyalgia** | |  |  |
| 2 | -98 470 | -100% | -98 400 -100% | | 11,89 | 0.988 |
| 3 | -87 062 | 11.6 | -86 954 11.6 | | 79,15,6 | 0.966 |
| 4 | -81 147 | 6.8 | -81 002 6.8 | | 16,72,7,5 | 0.957 |
| 5 | -77 908 | 4.0 | -77 726 4.0 | | 12,3,74,7,4 | 0.957 |
| 6 | -75 762 | 2.8 | -75 543 2.8 | | 2,3,13,73,2,7 | 0.960 |
|  |  |  | **Osteoarthritis** | |  |  |
| 2 | -170 279 | -100% | -170 210 -100% | | 72,28 | 0.987 |
| 3 | -151 223 | 11.2 | -151 118 | 11.2 | 67,16,17 | 0.987 |
| 4 | -140 251 | 7.3 | -140 110 | 7.3 | 8,66,15,11 | 0.989 |
| 5 | -135 779 | 3.2 | -135 600 | 3.2 | 51,8,16,15,10 | 0.936 |
| 6 | -131 919 | 2.8 | -131 704 | 2.9 | 16,7,52,3,14,8 | 0.943 |

**Supplementary Table 3**. Odds ratios (ORs) with 95% confidence intervals (CIs) for the association between education and the treatment trajectory classes of health care use in MSD patients overall and in the three diagnostic groups spine pain, fibromyalgia (FM) and osteoarthritis (OA). Multinomial logistic regression adjusted by age, sex and country background was used.

|  | **Education** | **No. of patients** | **Stable vs low use**  **OR (95% CI)** | **Descending vs low use**  **OR (95% CI)** | **High vs low use**  **OR (95% CI)** | **p-value** |
| --- | --- | --- | --- | --- | --- | --- |
| **All MSD** | **Elementary school** | 120 106 | 1.00 | 1.00 | 1.00 | 0.01 |
|  | **High school** | 218 917 | 1.01 (0.98-1.03) | 1.24 (1.21-1.28) | 1.31 (0.69-1.13) |  |
|  | **University** | 155 290 | 0.83 (0.81-0.85) | 1.34 (1.11-1.60) | 1.23 (1.26-1.35) |  |
| **Spine pain** | **Elementary school** | 23 319 | 1.00 | 1.00 | 1.00 | 0.01 |
|  | **High school** | 39 187 | 0.90 (0.86-0.97) | 1.30 (1.23-1.37) | 1.33 (1.23-1.43) |  |
|  | **University** | 29 808 | 0.71 (0.67-0.75) | 1.69 (1.60-1.79) | 1.33 (1.22-1.45) |  |
| **FM** | **Elementary school** | 3 306 | 1.00 | 1.00 | 1.00 | 0.03 |
|  | **High school** | 5 090 | 0.91 (0.79-1.05) | 1.21 (1.01-1.45) | 1.53 (1.22-1.93) |  |
|  | **University** | 3 778 | 0.63 (0.53- 0.74) | 1.53 (1.27-1.83) | 1.47 (0.16-1.88) |  |
| **OA** | **Elementary school** | 2 769 | 1.00 | 1.00 | 1.00 | 0.003 |
|  | **High school** | 5 147 | 1.23 (1.04-1.46) | 1.25 (1.09-1.44) | 1.42 (1.21-1.70) |  |
|  | **University** | 2 621 | 1.25 (1.02-1.53) | 1.37 (1.17-1.60) | 1.53 (1.28-1.84) |  |

|  | **Country background** | **No of patients** | **Stable vs low use**  **OR (95% CI)** | **Descending vs low use**  **OR (95% CI)** | **High vs low use**  **OR (95% CI)** | **p-value** |
| --- | --- | --- | --- | --- | --- | --- |
| **All MSD** | **Norway** | 398 033 | 1.00 | 1.00 | 1.00 | 0.002 |
|  | **Western countries** | 54 044 | 0.91 (0.88-0.94) | 0.85 (0.83-0.88) | 0.75 (0.72-0.78) |  |
|  | **Other** | 54 417 | 1.00 (0.97-1.03) | 0.53 (0.51-0.55) | 0.60 (0.58-0.63) |  |
| **Spine pain** | **Norway** | 69 303 | 1.00 | 1.00 | 1.00 | < 0.001 |
|  | **Western countries** | 11 454 | 0.94 (0.88-1.0) | 0.80 (0.75-0.85) | 0.79 (0.72- 0.87) |  |
|  | **Other** | 14 325 | 1.24 (1.14-1.35) | 0.44 (0.39-0.49) | 0.63 (0.54-0.74) |  |
| **FM** | **Norway** | 9 511 | *1.00* | *1.00* | *1.00* | < 0.001 |
|  | **Western countries** | 1 309 | *0.94 (0.77-1.14)* | *0.95 (0.77-1.18)* | *0.93 (0.71-1.23)* |  |
|  | **Other** | 1 701 | *0.50 (0.32-0.77)* | *0.29 (0.15-0.54)* | *0.44 (0.22-0.85)* |  |
| **OA** | **Norway** | 9571 | *1.00* | *1.00* | *1.00* | < 0.001 |
|  | **Western countries** | 705 | *0.85 (0.63-1.13)* | *0.95 (0.77-1.19)* | *0.89 (0.69-1.14)* |  |
|  | **Other** | 408 | *0.10 (0.01-0.72)* | *0.81 (0.46-1.44)* | *0.56 (0.26-1.22)* |  |

**Supplementary Table 4.** Odds ratio (ORs) with 95% confidence intervals of the association between country background and treatment trajectory classes of health care use, in MSD patients overall and by diagnostic groups spine pain, fibromyalgia (FM) and osteoarthritis (OA). Multinomial logistic regression was used.


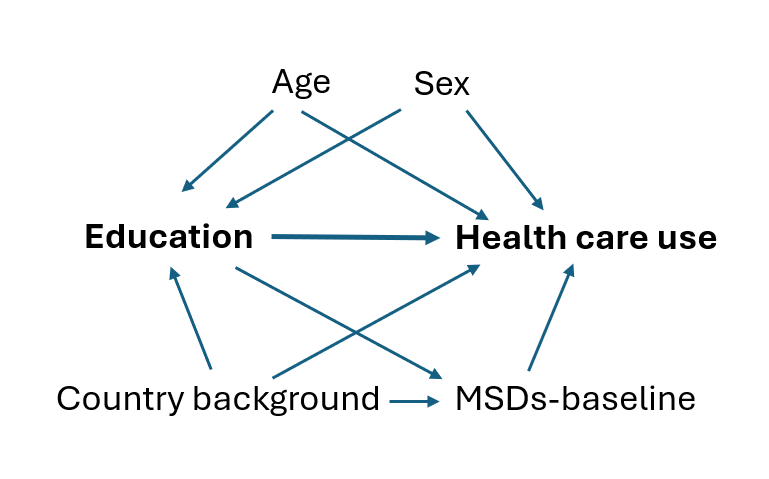


**Supplementary Figure 1**. Directed acyclic graph showing our assumptions about causal pathways between education (exposure of interest), other factors and health care use (outcome). We assumed causal pathways from education to the number of musculoskeletal disorders at baseline (MSDs-baseline) and from MSDs-baseline to health care use, hence MSDs-baseline is a mediator on the path from education to health care use. Age, sex and country background are known to affect education and health care use and are confounders. The minimal set of covariates necessary to adjust for in the multinomial regression analysis to estimate the total effect of education on health care use are sex, age and country background.


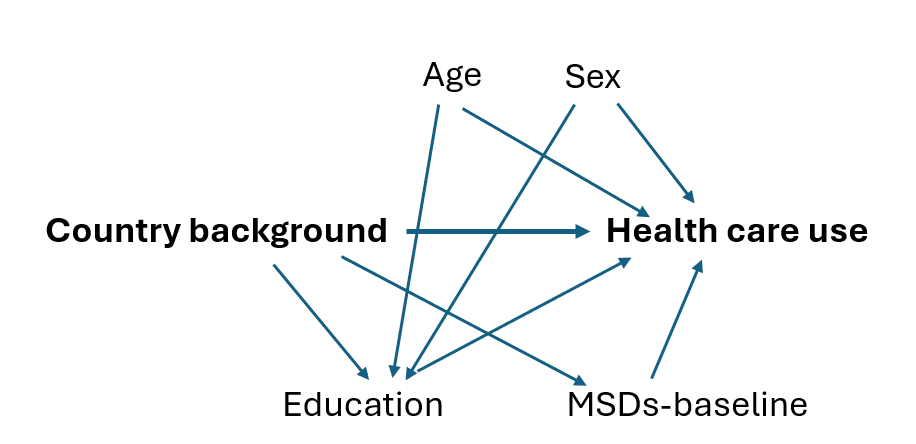


**Supplementary Figure 2.** Directed acyclic graph showing our assumptions about causal pathways between country background (exposure of interest), other factors and health care use (outcome). We assumed causal pathways from country background to education and the number of musculoskeletal diagnoses at baseline (MSDs-baseline) and from education and MSDs-baseline to health care use, hence education and MSDs-baseline are mediators on the causal path between country background and health care use. We assumed causal pathways from age and sex to health care use as they are known to affect education and health care use, but no causal path from age and sex to country background. Based on these assumptions no covariates are necessary to adjust for to estimate the total effect of country background on health care use.
