# Supplementary material for: Treatment trajectories among patients with musculoskeletal disorders in Norway – a register-based cohort study over 2 years
Source: Scand J Prim Health Care. 2026 Feb 25;44(1):2633751. doi: 10.1080/02813432.2026.2633751 (PMC12943818; doi:10.1080/02813432.2026.2633751)
Supplement: Supplementary_Article_2_june.docx [file IPRI_A_2633751_SM5596.docx]

Supplementary Table 1. Diagnostic groups according to the International Classification of Primary Care, second edition (ICPC-2) and the International Classification of Diseases 10^th^ revision (ICD-10).

|  | ICPC-2-  L-chapter | ICD-10  M-chapter |
| --- | --- | --- |
| Spine pain | 01-03, 83-86, | 40-54 |
| Fibromyalgia | 18 | 79.7 |
| Osteoarthritis | 89-91 | 15-19 |
| Shoulder/arm pain | 08,92 | 75 |
| Joint, bone and cartilage disorders | 05,07,09-13,15-17,20,70,88,94-95, 98-99 | 00-03,05-14,20-25,82-94 |
| Soft tissue disorders | 14,18-19,87,93 | 60-63,65-68,70-78 |
| Other | 04,26-29,71,73-82,96-97 | 30-36, 79 (-79.7), 95-96,99 |

Supplementary Table 2. Odds ratios (ORs) with 95% confidence intervals (CIs) for the association between education and the identified treatment trajectory classes of health care use in the musculoskeletal disorder (MSD) patients overall and in the three diagnostic groups spine pain, fibromyalgia (FM) and osteoarthritis (OA). Multinomial logistic regression adjusted by age, sex and country of origin was used.

|  | **Education** | **No. of patients** | **Stable vs low use**  **OR (95% CI)** | **Descending vs low use**  **OR (95% CI)** | **High vs low use**  **OR (95% CI)** | **p-value** |
| --- | --- | --- | --- | --- | --- | --- |
| **All MSD** | **Elementary school** | 120 106 | 1.00 | 1.00 | 1.00 | 0.01 |
|  | **High school** | 218 917 | 1.01 (0.98-1.03) | 1.24 (1.21-1.28) | 1.31 (0.69-1.13) |  |
|  | **University** | 155 290 | 0.83 (0.81-0.85) | 1.34 (1.11-1.60) | 1.23 (1.26-1.35) |  |
| **Spine pain** | **Elementary school** | 23 319 | 1.00 | 1.00 | 1.00 | 0.01 |
|  | **High school** | 39 187 | 0.90 (0.86-0.97) | 1.30 (1.23-1.37) | 1.33 (1.23-1.43) |  |
|  | **University** | 29 808 | 0.71 (0.67-0.75) | 1.69 (1.60-1.79) | 1.33 (1.22-1.45) |  |
| **FM** | **Elementary school** | 3 306 | 1.00 | 1.00 | 1.00 | 0.03 |
|  | **High school** | 5 090 | 0.91 (0.79-1.05) | 1.21 (1.01-1.45) | 1.53 (1.22-1.93) |  |
|  | **University** | 3 778 | 0.63 (0.53- 0.74) | 1.53 (1.27-1.83) | 1.47 (0.16-1.88) |  |
| **OA** | **Elementary school** | 2 769 | 1.00 | 1.00 | 1.00 | 0.003 |
|  | **High school** | 5 147 | 1.23 (1.04-1.46) | 1.25 (1.09-1.44) | 1.42 (1.21-1.70) |  |
|  | **University** | 2 621 | 1.25 (1.02-1.53) | 1.37 (1.17-1.60) | 1.53 (1.28-1.84) |  |

|  | **Country of origin** | **No. of patients** | **Stable vs low use**  **OR (95% CI)** | **Descending vs low use**  **OR (95% CI)** | **High vs low use**  **OR (95% CI)** | **p-value** |
| --- | --- | --- | --- | --- | --- | --- |
| **All MSD** | **Norway** | 398 033 | 1.00 | 1.00 | 1.00 | 0.002 |
|  | **Western countries** | 54 044 | 0.91 (0.88-0.94) | 0.85 (0.83-0.88) | 0.75 (0.72-0.78) |  |
|  | **Other** | 54 417 | 1.00 (0.97-1.03) | 0.53 (0.51-0.55) | 0.60 (0.58-0.63) |  |
| **Spine pain** | **Norway** | 69 303 | 1.00 | 1.00 | 1.00 | < 0.001 |
|  | **Western countries** | 11 454 | 0.94 (0.88-1.0) | 0.80 (0.75-0.85) | 0.79 (0.72- 0.87) |  |
|  | **Other** | 14 325 | 1.24 (1.14-1.35) | 0.44 (0.39-0.49) | 0.63 (0.54-0.74) |  |
| **FM** | **Norway** | 9 511 | *1.00* | *1.00* | *1.00* | < 0.001 |
|  | **Western countries** | 1 309 | *0.94 (0.77-1.14)* | *0.95 (0.77-1.18)* | *0.93 (0.71-1.23)* |  |
|  | **Other** | 1 701 | *0.50 (0.32-0.77)* | *0.29 (0.15-0.54)* | *0.44 (0.22-0.85)* |  |
| **OA** | **Norway** | 9571 | *1.00* | *1.00* | *1.00* | < 0.001 |
|  | **Western countries** | 705 | *0.85 (0.63-1.13)* | *0.95 (0.77-1.19)* | *0.89 (0.69-1.14)* |  |
|  | **Other** | 408 | *0.10 (0.01-0.72)* | *0.81 (0.46-1.44)* | *0.56 (0.26-1.22)* |  |

Supplementary Table 3. Odds ratio (ORs) with 95% confidence intervals (Cis) for the association between country of origin and the identified treatment trajectory classes of health care use, in musculoskeletal disorder (MSD) patients overall and by the diagnostic groups spine pain, fibromyalgia (FM) and osteoarthritis (OA). Multinomial logistic regression was used.


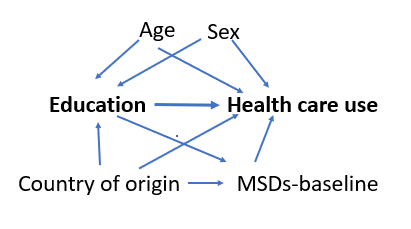


Supplementary Figure 1. Directed acyclic graph showing our assumptions about causal pathways between education (exposure of interest), other factors and health care use (outcome). We assumed causal pathways from education to the number of musculoskeletal disorders at baseline (MSDs-baseline) and from MSDs-baseline to health care use, hence MSDs-baseline is a mediator on the path from education to health care use. Age, sex and country of origin are known to affect education and health care use and are confounders. The minimal set of covariates necessary to adjust for in the multinomial regression analysis to estimate the total effect of education on health care use are sex, age and country of origin.


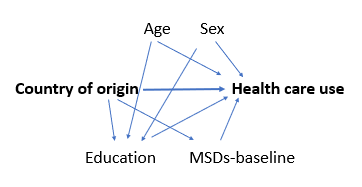


Supplementary Figure 2. Directed acyclic graph showing our assumptions about causal pathways between country of origin (exposure of interest), other factors and health care use (outcome). We assumed causal pathways from country of origin to education and the number of musculoskeletal disorders at baseline (MSDs-baseline) and from education and MSDs-baseline to health care use, hence education and MSDs-baseline are mediators on the causal path between country of origin and health care use. We assumed causal pathways from age and sex to health care use as they are known to affect education and health care use, but no causal path from age and sex to country of origin. Based on these assumptions no covariates are necessary to adjust for to estimate the total effect of country of origin on health care use.
